# Demenz und Migration

**DOI:** 10.1007/s00391-022-02078-8

**Published:** 2022-06-21

**Authors:** Jochen René Thyrian

**Affiliations:** Standort Rostock/Greifswald, Deutsches Zentrum für Neurodegenerative Erkrankungen (DZNE), Ellernholzstr. 1–2, 17489 Greifswald, Deutschland

Das Thema Demenz ist seit jeher ein zentrales Thema in den Alternswissenschaften und nicht zuletzt auch aufgrund des demografischen Wandels für die Gesellschaft relevant. Dem trägt die *Zeitschrift für Gerontologie und Geriatrie* in zahlreichen Facetten Rechnung, bereits seit Jahrzehnten. „Demenz“ rückte in den letzten Jahren auch zunehmend in das gesellschaftliche Bewusstsein. So befassen sich Bücher, Filme, Ausstellungen mit dem Thema; am Welt-Alzheimer-Tag am 21. September finden auch in Deutschland jedes Jahr vielfältige Aktionen statt, und die umliegenden Tage werden bereits vielerorts als Woche der Demenz geplant und organisiert. Die Forschung wurde mit spezifischen Förderprogrammen vorangetrieben (Leuchtturmprojekt Demenz, Zukunftswerkstatt Demenz u. Ä.), und eines der in den letzten Jahren gegründeten deutschen Zentren für Gesundheitsforschung fokussiert auf neurodegenerative Erkrankungen. Im Jahre 2020 wurde eine Strategie der (Demenz‑)Versorgungsforschung für die nächsten Jahre hier vorgestellt [1]. Nicht zuletzt auch aufgrund politischer Rahmenbedingungen wie der Formulierung einer nationalen Demenzstrategie scheint das Spektrum der Themen rund um die Demenz gut erschlossen zu sein. Migrationshintergrund wird in der Demenzforschung noch unzureichend, häufig nur als Kovariate betrachtet.

In diesem Schwerpunkt wird ein Aspekt beleuchtet, der bis dato wenig erforscht bzw. in vielerlei (Demenz‑)Forschung lediglich als Kovariate betrachtet wird – Migration bzw. Migrationshintergrund. Viele Menschen, die in den 1960er-Jahren nach Deutschland eingewandert sind, erreichen derzeit ein Alter, in dem die Wahrscheinlichkeit, an Demenz zu erkranken und pflegebedürftig zu werden, deutlich höher ist. In der Gesundheitsforschung gibt es einen Bereich, der sich explizit mit der Gesundheit von Menschen mit Migrationshintergrund beschäftigt. So zeigt sich z. B., dass Einstellungen zu Gesundheit, Zugang zu und Inanspruchnahme von medizinischen Leistungen/Hilfen, aber auch die Prävalenz bestimmter Gesundheitsprobleme mit einer Migrationsgeschichte assoziiert sein können. Spezifische Forschung zu Demenz und Migrationshintergrund in Deutschland ist rar, dabei geht man von schätzungsweise rund 100.000 Menschen mit Demenz mit Migrationshintergrund in Deutschland aus [4]. Dieser Schwerpunkt nähert sich deshalb dem Thema aus vier unterschiedlichen Richtungen an: Zugang zu Versorgung, Einfluss von Demenz auf Erst- und Zweitsprache, Einfluss von Migrationshintergrund auf herausforderndes Verhalten und konzeptionelle Einordnung des Themas in ein komplexeres Konstrukt.

In einem ersten Beitrag analysieren *Jessica Monsees et al.* mithilfe eines Scoping-Review, wo in Deutschland kultursensible Informationen, Versorgungsangebote, aber auch Projekte bekannt sind oder durchgeführt werden/wurden [5]. Dies stellt sicher keine umfassende Erhebung dar und kann nicht dahin interpretiert werden, dass es in Regionen, in denen kein Angebot gefunden wurde, solche nicht geben würde. Zum einen ist der Anteil an Menschen mit Migrationshintergrund mit Demenz regional unterschiedlich, zum anderen musste bei dem Review eine Auswahl an Informationsquellen getroffen werden. Nichtsdestotrotz wird eines auch hier schon deutlich – systematische Verfügbarkeit allein von entsprechender Information ist eine Herausforderung, regional geclustert, und Angebote sind wahrscheinlich nicht bedarfsgerecht vorhanden. Hier müssen differenziertere Analysen folgen.

Der Beitrag von *Katrin Bente Karl* [3] beleuchtet einen zentralen Unterschied zwischen Menschen mit und ohne Migrationshintergrund – die Muttersprache. In einer Fallstudie mit Datenmaterial, welches über 4 Jahre hinweg erhoben wurde, beschreibt sie mithilfe linguistischer Methoden die Veränderungen in der Erst- und Zweitsprache und erweitert eindrucksvoll unser Verständnis vom Zusammenspiel von Sprache und Demenz. Sie beschreibt in dem vorliegenden Fall eine stärkere Abnahme der Sprachkompetenz in der Erstsprache gegenüber der Zweitsprache, deren Niveau jedoch merklich über dem der Zweitsprache bleibt. Die Limitationen einer Kasuistik sind bekannt und werden auch hier im speziellen Fall diskutiert. Der Artikel weist jedoch darauf hin, dass in der Erforschung des Zusammenhangs zwischen Demenz und Sprache, gerade bei multilingualen Personen, noch vieles unklar ist.

Ein internationaler Beitrag von *Corina Bosma und Carolien Smits* [2] untersucht das mit einer Demenz assoziierte Symptom des herausfordernden Verhaltens bei Menschen mit Migrationshintergrund. Mithilfe von halb-strukturierten Interviews mit 20 erfahrenen, beruflich Pflegenden zeigen sie den möglichen Einfluss des Migrationshintergrundes auf die Symptomatik. Dabei ist es nicht Ziel der Analyse, Unterschiede zwischen Menschen mit Demenz mit vs. ohne Migrationshintergrund zu beschreiben. Eher geht es darum, dass Migrationshintergrund als ein Erklärungsansatz für die Symptomatik bei der Versorgung an Demenz erkrankter Menschen erkannt und genutzt werden kann. Die Schlussfolgerungen werden deshalb auch weiter gefasst und fragen nach einer interkulturellen Sensibilität als Teil einer personenzentrierten Herangehensweise in der Versorgung.

Schlussendlich befasst sich der Beitrag der INTERDEM-Task Force „dementia and migration“ mit der konzeptionellen Einbindung von Migrationshintergrund in das Konzept der Intersektionalität [7]. Intersektionalität als Konstrukt ist geeignet, verschiedene Kategorien, die eine Person definiert oder beschreibt, in Beziehung zueinander zu setzen, Überlappungen zu beschreiben und dadurch auch Bedarfe für die Versorgung oder auch Forschung zu beschreiben. Die Autor*innen beschreiben u. a. eindrücklich, wie die Betrachtung der Situation an Demenz erkrankter Menschen durch die intersektionale Brille zu einer Verbesserung der dem Individuum angepassten Versorgung führen kann. In diesem Beitrag geht es damit weit über die Betrachtung des Migrationshintergrunds als dominantes Identitätsmerkmal hinaus.

Durch den internationalen Beitrag wird die Komplexität des Themas deutlich. Demenz und Migration ist ein internationales Thema, und es gibt Ähnlichkeiten, aber auch massive Unterschiede zwischen Ländern. Einen ersten Versuch, das Thema auf europäischer Ebene vergleichbar zu machen, wurde mit einem EU-Atlas Demenz und Migration vorgelegt [6]. Wir stehen noch ziemlich am Anfang dieser Forschungsrichtung. Es beginnt allein in der adäquaten Beschreibung dessen, was untersucht wird. „Mensch mit Migrationshintergrund“ ist eine grobe Kategorie: kultureller Hintergrund, Ethnie, indigene Bevölkerung, Minderheit, Zuwanderer usw. – Wen meinen wir, wie spezifisch müssen wir sein? Bedarf/Problem aufgrund der Erkrankung oder aufgrund des Migrationshintergrundes – Wie muss die Strategie aussehen, Bedarfe zu erkennen und zu befriedigen? Wovon hängt der Einfluss des Migrationshintergrundes auf die demenzspezifische Gesundheitsversorgung ab – Grad der Integration, Bildung, Alter, soziales Netzwerk …? Wir hoffen, mit den vier Impulsen die Diskussion anzuregen und die wissenschaftliche Befassung mit dem Thema zu fördern.
